# An Investigation of the Long and Short Term Behavioral Effects of General Anesthesia on Pediatric Dental Patients With Autism

**DOI:** 10.3389/froh.2021.679946

**Published:** 2021-08-17

**Authors:** Janine Tran, Jung-Wei Chen, Larry Trapp, Laura McCormack

**Affiliations:** ^1^Dental Resident, Pediatric Dentistry, Loma Linda, CA, United States; ^2^Pediatric Dentistry, Loma Linda, CA, United States; ^3^Dental Anesthesiologist, Loma Linda, CA, United States; ^4^Private Practice Dentist, Pediatric Dentistry, Irvine, CA, United States

**Keywords:** autism (ASD), general anesthesia, behavior effect, long term follow up, short term follow-up

## Abstract

**Purpose:** The purpose of this study was to compare the incidence of short and long term adverse behavioral effects of general anesthesia (GA) in healthy vs. moderate to severe autistic (ASD) children.

**Methods:** Forty healthy and 37 ASD children, aged 3–17 years, undergoing GA for dental surgery participated in this study. Their anesthesia records were reviewed, and their parents answered telephone surveys to assess activity level, sleep disturbances, gastrointestinal disturbances, central nervous system effects, and respiratory depression. Three follow-up surveys were taken 8 h, 24 h, and 3 months post-surgery.

**Results:** Four hundred fifty-five incidences of adverse behavioral effects occurred within 8 h post-surgery. Significantly more ASD patients had difficulty walking (*P* = 0.016) and nausea (*P* = 0.030), while more healthy children snored in the car ride home (*P* = 0.036) and talked about the dental surgery (*P* = 0.027). Three months post-discharge, sixASD patients acted in a way that concerned caregivers compared to 0 healthy patients, (*P* = 0.008). Incidence of adverse behavioral effects significantly decreased from 8 to 24 h overall.

**Conclusions:** Most behavioral effects occur within 8 h post-surgery. There are potential long term adverse behavioral effects in ASD children from GA, but the chance is low and generally not long lasting.

## Introduction

Behavior guidance is an essential component of pediatric dentistry. When necessary, oral sedation is advised. When basic behavior guidance techniques and oral sedation are inadequate to achieve patient cooperation for treatment, general anesthesia (GA) becomes the main option. Very young or medically compromised patients require dental procedures performed under GA when they need more complicated or extensive treatment. There is ample research on the behavioral effects of oral sedation in healthy pediatric patients, but very few studies on the behavioral effects of GA.

McCormack et al. reported that most adverse behavioral events occurred within the first 8 h of discharge after oral sedation [1]. Ritwik et al. reported similar adverse effects 8 h, even up to 24 h after oral sedation [2]. These patients experienced irritability, vomiting, and increased sleep [2]. These studies could be applied to explore the behavioral effects of GA.

There are case reports, however, on behavioral changes in patients with autism spectrum disorder (ASD) after treatment under GA [3]. Matton et al. reported two patients born with ASD and attention deficit hyperactive disorder (ADHD) after undergoing GA for oral surgery [3]. The reported postoperative behavioral changes included irritability, reduced social engagement, loss of appetite, insomnia, and daytime somnolence [3]. Becker et al. concluded that 10–20% of hospitalized patients experience adverse anesthetic drug reactions [4]. As these are only case reports, a prospective cohort study would provide valuable insight into the behavioral effects of GA in this population of patients.

Many animal studies suggest that there is an association between early exposure to GA and long-term impairment of the developing cognition. Learning, memory, motor function, and attention could be affected [5–7]. Rodents and non-human primates exposed to anesthesia were found to experience apoptosis of neuronal cells, specifically in the cerebral cortex [8–12]. These studies tested the effects of ketamine, propofol, and isoflurane [3]. These anesthetics are N-methyl-D-aspartate glutamate receptor antagonists and GABA agonists, which may cause neurodegeneration in the cortical regions of the brain and may ultimately lead to disorders like ADHD and ASD [7].

There is an increasing concern regarding the potential effects of GA surgery in children. SmartTots is a partnership between the International Anesthesia Research Society and the Food and Drug Administration that funds clinical studies on the cognitive development in children exposed to GA at an early age [10, 13, 14]. Pediatric Anesthesia Neurodevelopment Assessment is a multicenter study that investigates the neurodevelopment in children who have undergone GA [15].

Existing research on the effects of GA on child behavior and cognitive development have inconclusive results. Behavioral regression was worse when the patient had pre-operative anxiety, was in pain, or had not eaten [3]. Camm et al. found that children who had dental treatment under GA were reported to have significantly more stress [16]. In addition, a study by Bakri et al. [5] concluded that children with at least two GA surgeries were at increased risk of developing anxiety, depression, ADHD, and sleep problems, but no increased risk for developing withdrawn or aggressive behaviors, fatigue, and pain.

The purpose of this study is to compare the incidence of short and long term adverse behavioral effects of GA in healthy vs. moderate to severe autistic children. Any changes in behavior will be assessed at the time of GA administration as well as 8 h, 24 h, and 3 months after discharge.

## Methods

The Loma Linda University (LLU) Institutional Review Board (IRB #5180244) approved this prospective study to recruit participants from the Koppel Special Care Dentistry (SCD) Surgery Center. Informed consent was obtained from the patients' legal guardian at the pre-operative exam. A total of 77 patients aged 3–17 years were selected for this study, with no restrictions on gender, race, or ethnicity. These patients have been referred for complete oral dental rehabilitation under GA due to uncooperative behavior that rendered them unable to undergo any dental treatment in a clinical setting. Also included were patients in whom oral sedation was or believed to be ineffective and referred to the SCD center. Group 1 included 40 healthy children to serve as the control group. Group 2 consisted of 37 patients with moderate to severe autism, with no other existing medical conditions. The patients in this group were levels 2 and 3 severity of the autism spectrum disorder, as defined by the *Diagnostic and Statistical Manual of Mental Disorders* [17]. Level two severity (“requiring substantial support”) is characterized by evident deficiencies in nonverbal and verbal communication skills, social impairments even with support, limited initiation of social interaction, diminished or abnormal responses, inflexible behavior, repetitive behaviors, and difficulty coping with change [1]. Level three severity (“requiring very substantial support”) includes the same criteria as Level two but more severe; the characteristics of a Level 3 autistic patient contributes to low-functioning behavior [17].

The standard procedure for dental surgery under GA was followed according to the protocol at the SCD surgery center as well as the American Dental Association (ADA) and American Academy of Pediatric Dentistry (AAPD) Guidelines. General anesthetics used were Ketamine via intramuscular injection or Sevoflurane via inhalation for induction and Propofol via intravenous (IV) delivery for maintenance. Other adjuvant agents may be provided as necessary, including opiates and non-steroidal anti-inflammatory drugs. Patients were continuously monitored, and their vital signs assessed with a precordial stethoscope, pulse oximeter, end tidal carbon dioxide, and electrocardiogram. Airway management was maintained by either intubated or non-intubated techniques. A treatment plan was created and reviewed with the legal guardian; subsequently, any and all necessary dental treatment was performed. Two percent lidocaine with 1:100,000 epinephrine was used as needed for local anesthesia, not to exceed 4 mg/kg lidocaine. When dental surgery was complete, patients were monitored in a recovery room, as defined by the ADA and AAPD Guidelines prior to discharge. These guidelines include continuous monitoring of vital signs, protective reflexes intact, the patient is able to talk and sit unaided if age appropriate, adequate hydration, and stable cardiovascular function and airway patency [18, 19]. Post-operative care instructions were given to the patients' legal guardian before discharge.

The investigator and research assistant interviewed the parents, using a script to reduce bias in the presentation of the survey questions. The research assistant was calibrated to provide the survey. A Spanish translator was hired to obtain informed consent and provide survey questions when needed. On the day of surgery, an initial survey was completed, including questions regarding patient behavior before and after surgery. Patient anesthesia records were used to answer questions regarding events during surgery. Telephone surveys were given to the patients' legal guardian 8 h, 24 h, and 3 months post-discharge. These time periods were selected based on previous studies which showed adverse behavioral events occurring within the first 8 h of discharge, even up to 24 h after oral sedation [1, 2]. Questions in the survey aimed to assess any changes (defined as adverse behavioral effects) in the patients' activity level, sleeping habits, gastrointestinal (GI) system, central nervous system (CNS), and respiratory depression. Paradoxical reactions were also measured by questions related to abnormally aggressive behavior, crying or screaming inconsolably, being hyperactive, and biting or scratching. Responses were either “yes” or “no,” and legal guardians were asked to explain any “yes” responses.

All responses were entered into Microsoft Excel 2016 (Microsoft, Inc., Redmond, Wash., USA) for data analysis. SPSS Statistics software, version 25 (IBM, Armonk, N.Y., USA) was used to obtain descriptive and inferential statistics to analyze the collected data. A Chi-squared test was used to determine if there was a significant difference in incidence of behavioral effects between the two groups and between the different time points when the surveys were taken. The *t*-test was used to evaluate the amount of drugs used during surgery and to evaluate the combined adverse effects compared between the two groups. A Pearson correlation analysis was performed to determine any association between age, gender, BMI, drug used during surgery, and combined adverse effects.

## Results

A total of 77 patients participated in this study, and the demographics data is summarized in Table 1. Fifty-seven patients (74%) received mask induction with Sevoflurane, while 20 (26%) received intramuscular induction with Ketamine. ASD patients had significantly more males, (*P* = 0.002), took more medications (*P* < 0.001), higher BMI (*P* < 0.001), and received intramuscular ketamine induction (*P* < 0.001). This group received significantly less lidocaine during procedure (*P* = 0.01). There was no significant difference between the two groups for post-surgical recovery time (*P* = 0.117). The more common drugs used during GA were Ketamine, Propofol, Ketorolac, Zofran, Demerol, Alfentanil, Decadron, Versed, Succinylcholine, Atropine, Remifentanil, and Benadryl. The healthy group received significantly higher dosages of Demerol (*P* = 0.001), Versed (*P* = 0.021), and Remifentanil (*P* = 0.001) (Table 1).

**Table 1:** Demographic data summary.

**Group**	**Male** ***N*** **(%)**	**Female** ***N*** **(%)**	**Mean age (years)**	**Mean BMI**	**Mean surgery time (min)**	**Mean recovery time (min)**	**Mean xylocaine amount (mL)**
**Demographics**							
Healthy	20 (50.0)	20 (50.0)	4.5 ± 0.9	16.1 ± 1.4	68.4 ± 21.0	35.6 ± 16.0	1.3 ± 0.7
ASD	31 (83.8)	6 (16.2)	9.0 ± 3.9	19.8 ± 4.67	58.1 ± 24.7	41.4 ± 15.7	0.8 ± 0.8
Total	51 (66.2)	26 (33.8)	6.7 ± 3.5	17.9 ± 3.8	63.4 ± 23.3	38.4 ± 16.0	1.0 ± 0.7
**Group**	**NKDA** ***N*** **(%)**	**Antibiotic** ***N*** **(%)**	**Seasonal** ***N*** **(%)**	**Multiple** ***N*** **(%)**	**Other** ***N*** **(%)**	
**Allergies**							
Healthy	30 (75.0)	1 (2.5)	5 (12.5)	2 (5.0)	2 (5.0)		
ASD	26 (70.3)	3 (8.1)	6 (43.2)	1 (2.7)	1 (2.7)		
Total	56 (72.7)	4 (5.2)	11 (14.3)	3 (3.9)	3 (3.9)		
**Group**	**None** ***N*** **(%)**	**Albuterol** ***N*** **(%)**	**OTC allergy** ***N*** **(%)**	**Multi-vitamins** ***N*** **(%)**	**Melatonin** ***N (%)***	**Valproic** ***N*** **(%)**	**Multiple** ***N*** **(%)**
**Medications**							
Healthy	23 (57.5)	5 (12.5)	4 (10.0)	7 (17.5)	0 (0)	0 (0)	1 (2.5)
ASD	13 (35.1)	1 (2.7)	3 (8.1)	2 (5.4)	2 (5.4)	1 (2.7)	13 (35.1)
Total	36 (46.8)	6 (7.8)	7 (9.1)	9 (11.7)	2 (2.6)	1 (1.3)	14 (18.2)
**Drug**	**Healthy** **number (%)**	**ASD** **number (%)**	**Dose for Healthy** **(mean ± SD mg/kg)**	**Dose for ASD** **(mean ± SD mg/kg)**	***P*** **-value**		
**Drugs used during surgery**							
Propofol	40 (100.0)	37 (100.0)	9.3 ± 7.8	7.6 ± 5.0	0.243		
Ketamine	14 (35.0)	27 (73.0)	2.2 ± 1.4	2.7 ± 1.4	0.275		
Ketorolac	39 (97.5)	33 (89.2)	0.5 ± 0.1	0.5 ± 0.3	0.777		
Zofran	38 (95.0)	31 (83.8)	0.4 ± 1.6	0.1 ± 0.2	0.430		
Demerol	32 (80.0)	23 (62.2)	1.6 ± 0.6	1.0 ± 0.5	0.001^*^		
Alfentanil	21 (52.5)	16 (43.2)	40.9 ± 18.0	29.3 ± 16.0	0.050		
Decadron	27 (67.5)	27 (73.0)	0.4 ± 0.7	0.2 ± 0.1	0.089		
Versed	6 (15.0)	22 (59.5)	0.1 ± 0.01	0.04 ± 0.01	0.021^*^		
Succinylcholine	2 (5.0)	4 (10.8)	0.5 ± 0.1	0.4 ± 0.2	0.840		
Atropine	4 (10.0)	1 (2.7)	0.01 ± 0.0	0.004 (no sd)	N/A		
Remifentanil	1 (2.5)	3 (8.1)	32.2 (no sd)	4.0 ± 0.9	0.001^*^		
Benadryl	4 (10.0)	8 (21.6)	0.7 ± 0.1	0.5 ± 0.2	0.164		

* *P < 0.05 using t-test to indicate difference in average dose of each drug used between Healthy and ASD groups. Bold values mean they are statistically significant*.

Overall, there were 77 survey questions and 5,929 responses, out of which 700 (11.8%) were “Yes” responses that indicated an incidence of an adverse behavioral effect (Table 2). Survey 1 had 15 questions, 1,155 total responses, and 5 (0.043%) “Yes” responses to show adverse effect during GA surgery. Survey 2 had 29 questions, 2,233 responses, and 455 (20.38%) “Yes” responses. Survey 3 had 27 questions, 2,079 responses, and 194 (9.33%) “Yes” responses. Survey 4 had 6 questions, 462 responses, and 46 (9.96%) “Yes” responses.

**Table 2:** Survey questions comparing incidence of adverse behavioral events after GA.

	**Yes** ***N*** **(%)**	**Healthy** ***N*** **(%)**	**ASD** ***N*** **(%)**	***P*** **-value chi-square**
**Survey 1: Initial survey**				
**Prior to GA induction, did the patient:**				
1P. Cry or scream inconsolably?	8 (10.4)	3 (7.5)	5 (13.5)	0.388
2P. Exhibit any aggressive behavior?	7 (9.1)	0 (0)	7 (18.9)	0.004^*^
3P. Bite or scratch anyone?	2 (2.6)	0 (0)	2 (5.4)	0.136
4P. Seem hyperactive?	8 (10.4)	2 (5.0)	5 (13.5)	0.107
5. Speak age-appropriately?	43 (55.8)	38 (95.0)	5 (13.5)	<0.001^*^
6. Walk on their own, if age appropriate?	76 (98.7)	39 (97.5)	37 (100)	0.333
7. Does your child snore?	21 (27.3)	13 (32.5)	7 (18.9)	0.113
**During GA surgery, did the patient:**				
8A. Require the use of reversal agents?	4 (5.2)	1 (2.5)	3 (8.1)	0.268
9A. Saturation level ever drop below 90%?	1 (1.3)	0 (0)	1 (2.7)	0.295
10A. Require head repositioning? (> five times per hour)	0 (0)	0 (0)	0 (0)	N/A
11A. Have any abnormal rash?	0 (0)	0 (0)	0 (0)	N/A
12A. At any point was treatment aborted? (if yes, why?)	0 (0)	0 (0)	0 (0)	N/A
**At discharge, could the patient:**				
13. Sit unaided?	77 (100)	40 (100)	37 (100)	N/A
14. Hold their head up on their own?	77 (100)	40 (100)	37 (100)	N/A
15. Speak age-appropriately?	43 (55.8)	38 (95.0)	5 (13.5)	<0.001^*^
**Survey 2: 8-H GA follow-up phone survey**				
**After leaving the surgery clinic, did your child:**				
1P. Cry or scream inconsolably?	12 (15.6)	8 (20.0)	4 (10.8)	0.267
2P. Exhibit any abnormally aggressive behavior?	4 (5.2)	4 (10.0)	0 (0)	0.048
3P. Bite or scratch anyone?	1 (1.3)	0 (0)	1 (2.7)	0.295
4P. Seem hyperactive?	7 (9.1)	4 (10.0)	3 (8.1)	0.773
5S. Fall asleep on the car ride home?	66 (85.7)	37 (92.5)	29 (7.8)	0.077
5a. Does your child normally sleep in the car?	36 (46.8)	26 (65.0)	10 (27.0)	0.001^*^
5bR. Did your child snore?	21 (27.3)	15 (37.5)	6 (16.2)	0.036^*^
5c. Does your child usually snore?	19 (24.7)	13 (32.5)	6 (16.2)	0.098
5dS. Was it difficult to awaken your child when you arrived home?	11 (14.3)	4 (10.0)	7 (18.9)	0.264
6U. Act in a way that made you concerned and caused you to pull the car over?	2 (2.6)	1 (2.5)	1 (2.7)	0.955
7S. Sleep soon after arriving home?	48 (62.3)	24 (60.0)	24 (64.9)	0.660
7aS. Did your child complain of bad dreams?	3 (3.9)	1 (2.5)	2 (5.4)	0.510
8L. Need help to sit up?	18 (23.4)	7 (17.5)	11 (29.7)	0.205
9L. Have difficulty walking?	27 (35.1)	9 (22.5)	18 (48.6)	0.016^*^
10L. Seem lethargic?	46 (59.7)	26 (65.0)	20 (54.1)	0.328
11L. Play immediately after arriving home?	19 (24.7)	12 (30.0)	7 (18.9)	0.260
12C. Talk less than normal or refuse to talk?	32 (41.6)	17 (42.5)	15 (40.5)	0.862
13C. Talk more than normal?	0 (0)	0 (0)	0 (0)	N/A
14C. Slur or speak incoherently?	5 (6.5)	3 (7.5)	3 (8.1)	0.573
15C. Complain of or seem dizzy?	23 (29.9)	8 (20.0)	15 (40.5)	0.070
16. Have any memory of what happened at the surgery clinic?	26 (33.8)	14 (35.0)	12 (32.4)	0.101
17. Talk about the dental surgery?	32 (41.6)	19 (47.5)	13 (35.1)	0.027^*^
18C. Have or complain of a headache?	4 (5.2)	1 (2.5)	3 (8.1)	0.167
19G. Complain of nausea?	10 (13.0)	2 (5.0)	8 (21.6)	0.030^*^
20G. Vomit?	9 (11.7)	3 (7.5)	6 (16.2)	0.234
21G. Have an upset stomach?	3 (3.9)	2 (5.0)	1 (2.7)	0.603
22G. Have diarrhea?	1 (1.3)	0 (0)	1 (2.7)	0.295
23U. Take any medication?	33 (42.9)	18 (45.0)	15 (40.5)	0.693
24U. Have any abnormal rash?	3 (3.9)	2 (5.0)	1 (2.7)	0.603
**Survey 3: 24-H GA follow-up phone survey**				
**After leaving the surgery clinic, did your child:**				
1P. Cry or scream inconsolably?	5 (6.5)	3 (7.5)	2 (5.4)	0.709
2P. Exhibit any abnormally aggressive behavior?	1 (1.3)	1 (2.5)	0 (0)	0.333
3P. Bite or scratch anyone?	0 (0)	0 (0)	0 (0)	N/A
4P. Seem hyperactive?	10 (13.0)	5 (12.5)	5 (13.5)	0.895
5S. Sleep more or less than normal?	19 (24.7)	9 (22.5)	10 (27.0)	0.645
5aR. Did your child snore?	17 (22.1)	12 (30.0)	5 (13.5)	0.081
5b. Does your child usually snore?	18 (23.4)	12 (30.0)	6 (16.2)	0.153
5cS. Was it difficult to awaken your child?	4 (5.2)	1 (2.5)	3 (8.1)	0.303
5dS. Did your child complain of bad dreams?	2 (2.6)	0 (0)	2 (5.4)	0.185
6L. Need help to sit up?	2 (2.6)	2 (5.0)	0 (0)	0.168
7L. Have difficulty walking?	3 (3.9)	2 (5.0)	1 (2.7)	0.603
8L. Seem lethargic?	17 (22.1)	9 (22.5)	8 (21.6)	0.926
9L. Play more or less than normal?	16 (20.8)	6 (15.0)	10 (27.0)	0.194
10C. Talk less than normal or refuse to talk?	6 (7.8)	2 (5.0)	4 (10.8)	0.342
11C. Talk more than normal?	6 (7.8)	3 (7.5)	3 (8.1)	0.921
12C. Slur or speak incoherently?	3 (3.9)	2 (5.0)	1 (2.7)	0.511
13C. Complain of or seem dizzy?	4 (5.2)	3 (7.5)	1 (2.7)	0.343
14. Have any memory of what happened at the surgery clinic?	17 (22.1)	8 (20.0)	9 (24.3)	0.078
15. Talk about the dental surgery?	25 (32.5)	15 (37.5)	10 (27.0)	0.048^*^
16C. Have or complain of a headache?	1 (1.3)	0 (0)	1 (2.7)	0.185
17G. Complain of nausea?	0 (0)	0 (0)	0 (0)	N/A
18G. Vomit?	0 (0)	0 (0)	0 (0)	N/A
19G. Have an upset stomach?	2 (2.6)	1 (2.5)	1 (2.7)	0.955
20G. Have diarrhea?	1 (1.3)	0 (0)	1 (2.7)	0.295
21U. Take any medication?	29 (37.7)	14 (35.0)	15 (40.5)	0.616
22U. Have any abnormal rash?	1 (1.3)	1 (2.5)	0 (0)	0.333
23U. Act in a way that made you concerned?	3 (3.9)	1 (2.5)	2 (5.4)	0.510
**Survey 4: 3-months GA follow-up phone survey**				
**Since the 24-h follow-up, did your child exhibit any:**				
1C. Psychological problems (aggressive behavior, crying or screaming, insomnia, nightmares)?	8 (10.4)	2 (5.0)	6 (16.2)	0.107
2G. Gastrointestinal problems (nausea, vomiting, diarrhea, constipation, upset stomach, loss of appetite)?	8 (10.4)	3 (7.5)	5 (13.5)	0.388
3C. Neurological problems (relapse in behavior, dizziness, or headaches)?	3 (3.9)	1 (2.5)	2 (5.4)	0.510
4P. Seem hyperactive?	14 (18.2)	4 (10.0)	10 (27.0)	0.053
5U. Act in a way that made you concerned?	6 (7.8)	0 (0)	6 (16.2)	0.008^*^
6U. Any changes in medications after surgery?	7 (9.1)	2 (5.0)	5 (12.5)	0.194

*N, Number of Responders; %, Frequency*,

* *P < 0.05 using chi-square test to indicate differences between Healthy and ASD who answered “Yes.” P, Paradoxical Reaction; S, Sleep Disturbance; R, Respiratory Effects; L, Activity Level; C, CNS Effects; G, GI Disturbance; A, Adverse Reaction During Surgery; U, Unusual Occurrence. Bold values mean they are statistically significant*.

**Figure 1:**
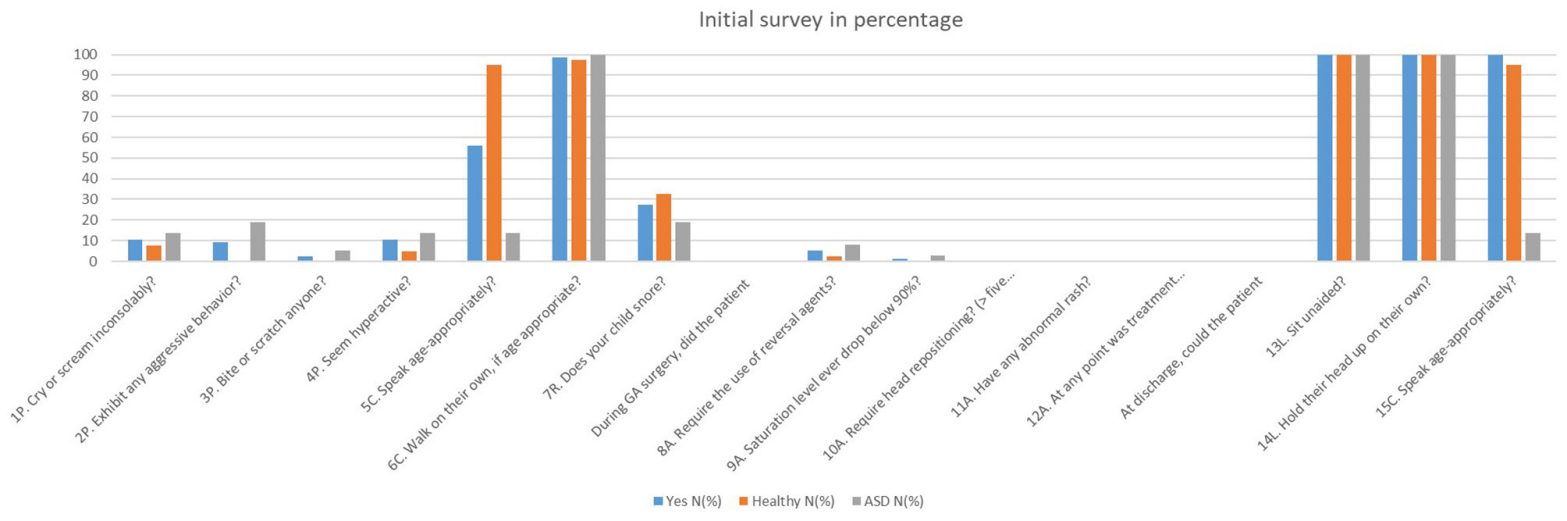
Initial survey in percentage.

**Figure 2:**
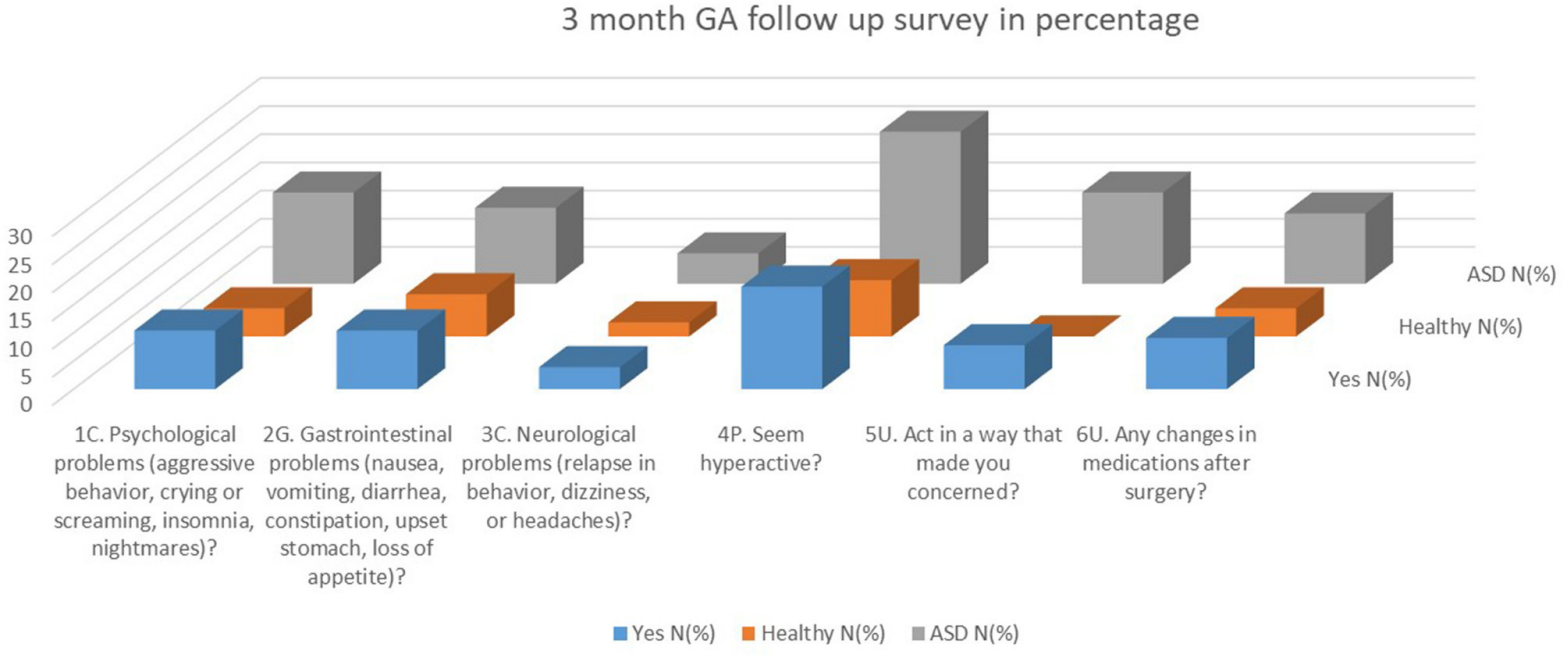
Three months FA follow up in percentage.

At pre-op survey 1 (Figure 1), there were significant differences in behavior prior to GA induction. Significantly more ASD patients exhibited aggressive behavior (*P* = 0.004). Significantly more healthy patients spoke age-appropriately prior to GA induction (*P* < 0.001) and at discharge (*P* < 0.001). At 8 h post-discharge, significantly more healthy patients normally slept in the car (*P* < 0.001), snored on the car ride home (*P* = 0.036), and talked about the dental surgery (*P* = 0.027). Significantly more ASD patients had difficulty walking (*P* = 0.016) and complained of nausea (*P* = 0.030) at this time period (Table 2). At 24 h post-discharge, significantly more healthy patients talked about the dental surgery (*P* = 0.048). At 3 months post-surgery (Figure 2), significantly more ASD patients acted in a way that concerned their parent/caregiver (*P* = 0.008). According to the Fisher's exact test, there was significantly more hyperactivity in the ASD group (*P* = 0.05) during this time. For the remaining survey questions, there were no significant differences between the two groups.

Questions were grouped together to compare types of adverse behavioral effects between the healthy and ASD groups (Table 2). There was no significant difference in the occurrence of an adverse reaction during surgery (*P* = 0.143). Comparing pre-operative behavior, significantly more ASD patients showed aggressive behavior and cried inconsolably (*P* = 0.008) and did not speak appropriately for their age (*P* < 0.001). There was no significant difference in incidence of paradoxical reactions at 8 h after (*P* = 0.174) and 24 h after (*P* = 0.189). Using Fisher's exact test, the ASD group had significantly more paradoxical reactions (*P* = 0.05), reported as more hyperactivity, at 3 months post-surgery. Significantly more healthy patients reported respiratory effects (*P* = 0.032) at 8 h post-discharge, specifically snoring in the car ride home and usually snoring in general; there was no difference between the two groups at 24 h post-discharge for this category. At 3 months post-surgery, significantly more ASD patients reported GI disturbance (*P* = 0.008) and unusual occurrences (*P* = 0.013) in which they behaved in a way that concerned parents/caregivers. There were no significant differences in CNS effects, level of activity, or sleep disturbance in any of the surveys. However, there was a trend in the 24-h survey showing more ASD patients with difficulty waking up and complaints of bad dreams (*P* = 0.062).

The paired *t*-test was used to determine any significant difference between the specific adverse effect at 8 h vs. 24 h after discharge (Table 3). The Pearson correlation test demonstrated that significant correlation between particular categories of adverse behavioral effect (Table 4). All pairs of adverse effects listed in Table 4 had a positive correlation.

**Table 3:** Paired *t*-test statistics for adverse behavioral effects over time.

**Survey questions**	**Mean**	**Standard deviation**	**Standard error**	**Significance**
Paradoxical reaction 8 h	0.31	0.59	0.07	*P* = 0.219
Paradoxical reaction 24 h	0.21	0.5	0.06	Not significant
Sleep disturbance 8 h	0.17	0.45	0.05	***P*** = 0.034^*^
Sleep disturbance 24 h	0.08	0.27	0.03	**Significant decrease**
Level of activity 8 h	1.43	0.98	0.11	***P*** < 0.001^*^
Level of activity 24 h	0.49	0.9	0.1	**Significant decrease**
CNS effects 8 h	1.71	1.24	0.15	***P*** < 0.001^*^
CNS effects 24 h	0.87	0.97	0.12	**Significant decrease**
GI discomfort 8 h	0.3	0.62	0.07	***P*** = 0.001^*^
GI discomfort 24 h	0.04	0.25	0.03	**Significant decrease**
Respiratory effects 8 h	0.52	0.77	0.09	*P* = 0.228
Respiratory effects 24 h	0.45	0.79	0.09	Not significant
Unusual occurrence 8 h	0.45	0.53	0.06	*P* = 0.581
Unusual occurrence 24 h	0.42	0.57	0.06	Not significant
Unusual occurrence 24 h	0.41	0.57	0.06	***P*** = 0.001^*^
Unusual occurrence 3 months	0.17	0.44	0.05	**Significant decrease**
Unusual occurrence 8 h	0.45	0.52	0.06	***P*** < 0.001^*^
Unusual occurrence 3 months	0.17	0.44	0.05	**Significant decrease**

*N = 77 patients*.

* *P < 0.05 using paired t-test to indicate significant change in incidence of effects between time points. Reported are significant decreases from earlier time point to later time point. Bold values mean they are statistically significant*.

**Table 4:** Correlation between adverse behavioral effect type.

**Adverse effect**	**Correlated with (r, *P*-value)**
CNS at 8 h	Paradoxical at 24 h (r = +0.290, *P* = 0.015) CNS at 24 h (r = +0.512, *P* < 0.001) Sleep at 8 h (r = +0.290, *P* = 0.016) Sleep at 24 h (r = +0.491, *P* < 0.001) Activity level at 8 h (r = +0.570, *P* < 0.001) Activity level at 24 h (r = +0.510, *P* < 0.001) GI discomfort at 8 h (r = +0.433, *P* < 0.001) Unusual occurrence at 8 h (r = +0.281, *P* = 0.017) Unusual occurrence at 3 months (r = +0.278, *P* = 0.021)
CNS at 24 h	Paradoxical reaction at 24 h (r = +0.336, *P* = 0.004) Sleep disturbance at 8 h (r = +0.358, *P* = 0.002) Sleep disturbance at 24 h (r = +0.363, *P* = 0.002) Activity level at 24 h (r = +0.426, *P* < 0.001) Unusual occurrence at 8 h (r = +0.275, *P* = 0.021) Unusual occurrence at 24 h (r = +0.305, *P* = 0.010) Unusual occurrence at 3 months (r = +0.451, *P* < 0.001)
CNS at 3 months	Paradoxical reaction at 8 h (r = +0.246, *P* = 0.031) Paradoxical reaction at 24 h (r = +0.254, *P* = 0.026) Unusual occurrence at 3 months (r = +0.395, *P* < 0.001)
GI discomfort at 8 h	Pre-op behavioral issue (r = +0.346, *P* = 0.002) Sleep disturbance at 24 h (r = +0.271, *P* = 0.010) Activity level at 8 h (r = +0.259, *P* = 0.023) Activity level at 24 h (r = +0.411, *P* < 0.001)
Unusual occurrence at 24 h	Sleep disturbance at 24 h (r = +0.439, *P* < 0.001) GI discomfort at 24 h (r = +0.246, *P* = 0.031) Unusual occurrence at 8 h (r = +0.404, *P* < 0.001) Unusual occurrence at 24 h (r = +0.231, *P* = 0.043)
Sleep disturbance at 24 h	Pre-op behavioral issue (r = +0.294, *P* = 0.010) Paradoxical reaction at 24 h (r = +0.281, *P* = 0.015) Activity level at 8 h (r = +0.272, *P* = 0.018)
Respiratory effect at 8 h	Respiratory effect at 24 h (r = +0.518, *P* < 0.001)
Activity level at 8 h	Activity level at 24 h (r = +0.295, *P* = 0.009)

*Significance set at P < 0.05. Listed are the significant correlation between specific type of adverse behavioral effect*.

There was a slightly higher incidence of neurological problems with Ketorolac use during surgery (*P* = 0.054). There was a significantly higher incidence of behavior that concerned parents/caregivers (*P* = 0.031) and a slightly higher incidence of GI discomfort (*P* = 0.051) with Demerol use. Versed use had significantly higher incidence of psychological problems (*P* = 0.016) and changes in medications after surgery (*P* = 0.043). Atropine use had significantly higher incidence of psychological problems (*P* = 0.025). Remifentanil use had significantly higher incidence of GI discomfort (*P* = 0.008) and changes in medications after surgery (*P* = 0.003). Benadryl use was significantly higher with no changes in medications after surgery (*P* = 0.039).

The ASD group had a larger age range: 11 were younger than 6 years, 18 were 6 years to <12 years, and eight were 12 years and older. Significantly higher doses of ketamine (*P* = 0.048), ketorolac (*P* = 0.021), and versed (*P* = 0.021) were used in the 6 to <12-years group. Significantly more patients in this group reported that they usually snored (*P* = 0.023). In the 8-h survey, there were significantly fewer patients in the 6 to <12-years group that normally slept in the car (*P* = 0.003) and had GI discomfort (*P* = 0.044). Significantly more patients in this group had CNS effects during this time period. In the 24-h survey, significantly fewer patients in the 6 to <12-years group cried or screamed inconsolably (*P* = 0.022) and had a memory about the dental surgery (*P* = 0.047); significantly more of these patients snored (*P* = 0.047). In the 3-months survey, significantly fewer patients in the 6 to <12-years group reported hyperactivity (*P* = 0.005). There were no significant differences in any age group for any other category.

## Discussion

The results of this study confirmed the statistics of children with ASD previously reported in the literature [15]. Specifically there were significantly more males and higher BMI in the ASD group. Significantly fewer ASD patients did not speak age-appropriately, which was expected because the inclusion criteria for the ASD group in this study was that the patient must have deficiencies in nonverbal and verbal communication skills. This group also had a greater tendency to show aggressive behavior at baseline. These types of behavior often render the use of intramuscular Ketamine induction because the patient is unable to cooperate for mask induction, which was demonstrated by the results of this study. The ASD group had a wider age range compared to the healthy group because of the patients available at SCD. When comparing demographics and responses for the three age groups, there were few significant differences most likely due to the fact that the majority of the ASD patients were between age 6 and 12 years.

A greater number of healthy patients reported snoring on the car ride home, which matched their positive response to regularly snoring in general. A larger number of healthy patients also talked about the dental surgery, most likely because the ASD patients had deficiencies in verbal communication. Patients in the ASD group had more nausea and difficulty walking at 8 h post-discharge, suggesting that this group experienced a stronger effect on GI disturbance and activity level overall. Patients who experienced CNS effects at 8 h post-discharge generally had higher incidence of CNS effects, sleep disturbances, and low activity level at 24 h post-discharge. More adverse behavioral effects can be expected at 24 h if the patient demonstrated CNS problems at 8 h post-discharge. The fact that adverse behavioral effects occurred at a 6.57% frequency during the 3-months survey showed that most of these effects last short term. Overall, most incidence of adverse behavioral effects significantly decreased over time (Table 2).

McCormack et al. concluded that a higher incidence of adverse effects occurred within 8 h after discharge [1]. In particular, there was increased sleep time, effect on activity level (difficulty walking, talking less than normal after arriving home), and CNS effects (slurring, difficulty speaking) [1]. Similarly, our study found that the greatest incidence of adverse behavioral effects occurred within 8 h after discharge, and the second highest incidence occurred 24 h after discharge. Between the 8-h and 24-h surveys in this study, there were significant decreases in sleep disturbance, effects on activity level, CNS effects, and GI discomfort. There were significant decreases in unusual occurrences (abnormal rash, changes in medications, and concerning behavior) between 8 h and 3 months and between 24 h and 3 months, suggesting that these effects are not long term (Table 3).

The 3-months follow up survey served to study the long-term effects of GA (Figure 2). Patients with aggressive behavior and hyperactivity at baseline tended to have higher incidence of adverse reactions during surgery, GI disturbances 8 h post-discharge, sleep disturbance 24 h post-discharge, and CNS effects 3 months post-discharge. This 3-months survey revealed that significantly more ASD patients acted in a way that concerned their parents/caregivers. One parent reported that the patient had a calmer and less active behavior with delayed response to commands. Several parents reported insomnia, random screaming, and more aggressive behavior. One patient was irritated more easily for the first month after surgery, but returned to their usual behavior the following month. Another patient in this group was newly diagnosed with ADHD. Previous studies suggested that certain general anesthetics may cause neurodegeneration of the brain, which may lead to ADHD and autism [7]. However, the patient in this study could have already been in the process of being diagnosed for ADHD. As Matton et al. reported, the GA could have caused oxidative stress on the brain [3]. This stress could contribute to post-surgery behavioral changes, including irritability, insomnia, and decreased social engagement, all of which were reported during the 3-months survey [3].

Each drug used during surgery was compared to responses in the 3-months survey to determine any potential long-term effects. Based on our study, the incidence of psychological problems, such as aggressive behavior, screaming, and crying may be associated with the use of Versed and Atropine during surgery. GI discomfort may be associated with Remifentanil and Decadron. Changes in medications after surgery may be associated with the use of Remifentanil and Versed, whereas Benadryl was associated with no changes in medications. Demerol is important to note because its use during surgery correlated with behavior that concerned parents and caregivers. Providers should be cautious with the long-term effects of these drugs during GA.

Although these findings shed light on the potential adverse behavioral effects of GA, this study is not without its limitations. The parent/caregiver's interpretation of the survey question may have biased their answer. More specifically, parents of the patients with ASD had difficulty answering certain questions because their child was nonverbal. Depending on how the investigators presented the question, the parent may have responded differently. There were different dental anesthesiologists providing the GA for this study. Although they all followed the guidelines set by the ADA and AAPD, each had their own combination of drugs they preferred to use. Such varying combination of drugs may have affected the outcomes of this study. The long-term follow up survey had a natural limitation. Responses may have been inaccurate as it could be difficult to remember behavior over a long period of time. Future research should reduce confounding factors, perhaps by reducing the patient age range or by creating more specific questions that would be easier to respond for nonverbal patients. Finally, the follow up time could be extended to 6 months post-discharge.

## Conclusion

Based on the results of this study, the following conclusions can be made:

1. Most adverse behavioral effects (i.e., aggressive behavior, crying, screaming, insomnia, nightmares) occurred at 8 and 24 h after GA.
2. There were significantly more adverse post-GA complications in moderate to severe ASD patients compared to healthy patients, especially at 8-h post-surgery.
3. At the 3-months follow up survey, significantly more ASD children behaved in a way that concerned their parents, mainly with reports of increased aggressive behavior, irritation, and screaming.
4. Significantly more ASD children received IM ketamine induction for GA.
5. Certain drugs used during GA surgery may have long-term effects. Versed and Atropine were associated with behavioral problems effects (i.e., aggressive behavior, crying, screaming, insomnia, nightmares), Remifentanil and Decadron with GI disturbances, and Demerol with occurrence of behavior that concerned parents.

## Data Availability Statement

The original contributions presented in the study are included in the article/supplementary material, further inquiries can be directed to the corresponding author/s.

## Author Contributions

JT collected data and drafted the manuscript. J-WC designed the study, supervised data collection, analyzed data, and reviewed the manuscript. LT supervised data collection and reviewed the manuscript. LM designed the survey and edited the manuscript. All authors contributed to the article and approved the submitted version.

## Conflict of Interest

The authors declare that the research was conducted in the absence of any commercial or financial relationships that could be construed as a potential conflict of interest.

## Publisher's Note

All claims expressed in this article are solely those of the authors and do not necessarily represent those of their affiliated organizations, or those of the publisher, the editors and the reviewers. Any product that may be evaluated in this article, or claim that may be made by its manufacturer, is not guaranteed or endorsed by the publisher.
